# Eight versus twelve weeks of sofosbuvir-velpatasvir in treatment-naïve non-cirrhotic patients with chronic hepatitis C virus infection: Study protocol for a multicentric, open labelled, randomized, non-inferiority trial (RESOLVE trial)

**DOI:** 10.1371/journal.pone.0285725

**Published:** 2023-05-18

**Authors:** Ashish Awasthi, Harshita Katiyar, Sumit Rungta, Amar Deep, Vinod Kumar, Shalimar, Ajay Kumar, Prachi Tiwari, Amit Goel

**Affiliations:** 1 Centre for Chronic Disease Control, New Delhi, India; 2 Centre for Chronic Conditions and, Injuries, Public Health Foundation of India, Gurgaon, India; 3 Department of Hepatology, Sanjay Gandhi Postgraduate Institute of Medical Sciences, Lucknow, India; 4 Department of Gastroenterology, King George Medical University, Lucknow, India; 5 Department of Gastroenterology, Institute of Medical Sciences, Banaras Hindu University, Lucknow, India; 6 Department of Gastroenterology and Human Nutrition, All India Institute of Medical Sciences, New Delhi, India; 7 Department of Gastroenterology, Govind Ballabh Pant Institute of Postgraduate Medical Education and Research, New Delhi, India; Kaohsiung Medical University, TAIWAN

## Abstract

**Background:**

Hepatitis C virus (HCV) is a common cause of liver cirrhosis and hepatocellular carcinoma. Globally, nearly 71 million people have chronic HCV infection, and approximately 399,000 dies annually. In patients without cirrhosis, HCV infection is treated with 12 weeks of sofosbuvir/velpatasvir combination. Results from available small, single-centre observational studies suggest that the sofosbuvir/velpatasvir combination given for 8 weeks may be as effective as the standard 12 weeks of treatment. We propose to compare the treatment response of 12 weeks versus 8 weeks of sofosbuvir/velpatasvir in non-cirrhotic people with chronic HCV infection.

**Methods:**

This multicentric, randomized, open-label, non-inferiority trial will include 880 (2 arms x 440) treatment naïve, viraemic (HCV RNA >10,000 IU/mL), non-cirrhotic adults (age >18 years) with chronic hepatitis C. People who are at high-risk for HCV reinfection such as haemophiliacs, people who inject drugs, those on maintenance hemodialysis or having HIV will be excluded. The presence or absence of cirrhosis will be determined with a combination of history, examination, ultrasound, liver stiffness measured with transient elastography, APRI, FIB-4, and esophagogastroduodenoscopy. Participants will be randomized to receive either 8- or 12-week sofosbuvir/velpatasvir treatment. A blood specimen will be collected before starting the treatment (to determine the HCV genotype), after 4 weeks of treatment (for early virological response), and at 12 weeks after treatment discontinuation for SVR12.

**Discussion:**

The study will provide data on the efficacy of 8 weeks of treatment as compared to the standard of care (12 weeks) in non-cirrhotic patients with chronic HCV infection. Treatment for a shorter duration may improve treatment compliance, reduce the cost of treatment, and ease the treatment implementation from a public health perspective.

**Trial registration:**

Registered with Clinical Trial Registry of India (http://ctri.nic.in) Registration No. CTRI/2022/03/041368 [Registered on: 24/03/2022]—Trial Registered Prospectively.

## Introduction

### Background and rationale {6a}

Hepatitis C (HCV) virus is a hepatotropic virus which infects and multiplies in hepatocytes. A large proportion of those who acquire HCV infection fails to clear the virus naturally and progress to develop chronic HCV infection [1]. Unchecked HCV hepatitis, in a sizeable proportion, may progress to liver cirrhosis and hepatocellular carcinoma [2], both of which are potentially fatal conditions. The HCV infection is treated with orally acting drugs called direct-acting antivirals (DAAs). These DAAs are effective against certain genotypes (genotype-specific regimen) or all genotypes (pan-genotypic regimen) [3].

Globally, an estimated 71 million people live with HCV infection, accounting for approximately 399,000 annual deaths [4]. Pondering the huge public health importance of HCV-related morbidity and mortality, the World Health Assembly endorsed the Global Health Sector Strategy (GHSS) for 2016–2021 on viral hepatitis, which proposes to eliminate viral hepatitis as a public health threat by 2030 [5].

Our recent work systematic review of Indian data on HCV seroprevalence showed that it ranges between 0.4% and 1.0% in the country [6]. We also estimated that approximately 5.0 million people, not belonging to the groups at high risk of acquiring HCV infection, are anti-HCV positive [7]. Over 88% of the HCV burden in India is buried in low-risk general population [7]. Unsafe injections are the most common route of HCV transmission in these low-risk general population [8].

To abide with the targets set by WHO to achieve the elimination of HCV, Government of India has recently launched a public-funded program named “National Viral Hepatitis Control Program (NVHCP)”. However, the wider objective of the program is to prevent and control all viral hepatitis (Hepatitis A, Hepatitis B, Hepatitis C, and Hepatitis E) but it focuses primarily on HCV. The national guidelines for laboratory testing, diagnosis, and treatment are already in place, and the treatment has been started for a large number of patients.

Data from disease modelling has shown that the mass treatment of HCV will be cost-effective and cost saving [9]. Further, at the current costs, the pan-genotypic anti-HCV regimen is cost neutral to genotype-specific regimen [10]. The cost-effectiveness may further be enhanced if the treatment duration can be reduced from 12 to 8 weeks.

HCV infection is treated with DAAs. These DAAs are orally administered and are highly safe and effective. Sofosbuvir/velpatasvir for 12 weeks is the most widely used DAAs for HCV treatment. This combination successfully clears the virus in ~95% in the non-cirrhotic population. One of the major bottlenecks in successful HCV treatment is the need for “12 weeks duration” of treatment. Long-duration of treatment poses logistic issues of maintaining the drug supply, poor drug compliance, and increased cost of therapy. Hence, efforts are made to reduce the treatment duration without compromising effectiveness.

Few small observational studies, which have used different DAA combinations, have shown that the treatment outcome with 8 weeks of duration is comparable to 12 weeks of therapy [11–17]. Till date, we do not have any randomized controlled trial comparing the 8 versus 12 weeks of sofosbuvir/velpatasvir combination for HCV treatment. The results of the proposed study may reduce the treatment duration as well as the cost of therapy.

### Objectives {7}

#### Hypothesis

The duration of anti-HCV treatment can be reduced from 12 weeks to 8 weeks.

#### Research question

Can anti-HCV treatment duration be reduced from 12 weeks to 8 weeks without compromising the virological response?

#### Primary objective

To compare the sustained virological response at week 12 (SVR12) after stopping 8 weeks or 12 weeks of sofosbuvir/velpatasvir in non-cirrhotic patients with chronic HCV infection.

#### Secondary objective

To compare the proportion of participants completing their planned 8 weeks or 12 weeks of treatment.

#### Trial design {8}

Multicentric, Open-label, Randomized, Non-inferiority trial.

## Methods: Participants, interventions and outcomes

### Study setting {9}

Five tertiary care teaching hospitals located in various parts of India will participate in the study for data collection. Each of the participating centres will enrol at least 80 participants. Total sample size is 880 participants (440 x 2 arms). The participating centres are (i) Department of Gastroenterology, King George Medical University, Lucknow, India (www.kgmu.org) (ii) Department of Gastroenterology, Sanjay Gandhi Postgraduate Institute of Medical Sciences, Lucknow, India (www.sgpgims.ac.in) (iii) Department of Gastroenterology, Institute of Medical Sciences, Banaras Hindu University, Lucknow, India (www.bhu.ac.in/ims/) (iv) Department of Gastroenterology and Human Nutrition, All India Institute of Medical Sciences, New Delhi, India (www.aiims.edu) and (vi) Department of Gastroenterology, Govind Ballabh Pant Institute of Postgraduate Medical Education and Research, New Delhi, India (http://gbpant.delhigovt.nic.in).

Our protocol is registered with Clinical Trial Registry of India (http://ctri.nic.in; CTRI/2022/03/041368).

The data analysis will be conducted by the Centre for Chronic Conditions and, Injuries, Public Health Foundation of India, Gurgaon, India (www.phfi.org/iiph-delhi/).

### Eligibility criteria {10}

#### Participant selection

People with chronic HCV without cirrhosis who attend the outpatient services in either of the five participating institutes will be prospectively screened for the eligibility criteria and will be enrolled after obtaining written informed consent if found eligible. Each prospective participant will be screened only once for the eligibility criteria. They not be rescreened after an interval.

#### Inclusion criteria

1. Age >18 years
2. HCV mono-infection
3. Detectable HCV RNA (>10,000 IU/mL) in serum
4. Chronic HCV infection
5. No evidence of cirrhosis

#### Exclusion criteria

1. Presence of cirrhosis
2. HBsAg or HIV co-infection
3. Estimated GFR <30 ml/ minute
4. Prior exposure to DAAs
5. Any ongoing (active) or prior malignancy including hepatocellular carcinoma
6. Portal vein thrombosis
7. High-risk population such as people living with HIV (PLHIV), people on maintenance hemodialysis (MHD), thalassaemic or haemophiliacs, people who inject drugs, men have sex with men, high risk sexual behaviour
8. Acute hepatitis illness including alcoholic hepatitis
9. Presence of concomitant liver diseases such as autoimmune hepatitis, primary biliary cholangitis etc. Participants will be evaluated for concomitant liver diseases only if indicated on the basis of clinical features of laboratory investigations.
10. Participants on medication which are contraindicated to administer sofosbuvir/velpatasvir.
11. Participants on medication which have unavoidable potential drug to drug interaction with sofosbuvir/velpatasvir

Every person, infected with HCV and meeting our eligibility criteria, will be assumed to have chronic infection. Acute infection with HCV is very difficult to determine because most of acute infection remain asymptomatic. Further acute HCV are reported in high-risk population such as PLHIV, people on MHD, etc and we have excluded all those at high-risk for HCV infection. In present era of DAA, treatment and response of acute HCV are similar to that in chronic HCV.

Presence of absence of cirrhosis will be determined with a combination of combination of history, examination, ultrasound, Transient elastography, APRI, FIB-4, esophagogastroduodenoscopy (OGD).

“Presence of cirrhosis’ will be concluded if the participants have any of the following (i) clinical features of decompensation such as jaundice, ascites, hepatic encephalopathy or variceal bleed (ii) OGD, if available, showed esophageal or gastric varices (iii) Liver stiffness value (Fibroscan) >12.5 KPa or (iv) APRI is >2.0 PLUS FIB-4 >3.25, if Fibroscan is not available.

’Absence of cirrhosis’ shall be concluded if the participants have all of the following (i) no clinical feature of decompensation (ii) no varices in OGD, if performed (iii) TE <12.5 OR APRI <2.0 plus FIB-4 <3.25, if Fibroscan is not available. If there are discrepancies between Fibroscan and APRI/FIB-4, then the Fibroscan value shall be accepted for diagnosis.

#### Who will take informed consent? {26a}

Written informed consent will be taken after giving relevant trial information in the form of patient information documents (PID). The PID and the consent form will be printed in the mother tongue (Hindi) of the participants. All the participating study centres are located in the northern part of the country, where the mother tongue is Hindi. Written consent will be taken by either the principal investigators of the study site or the research staff after the counselling of the participants by the principal site investigator.

#### Additional consent provisions for collection and use of participant data and biological specimens {26b}

The PID contain information about the blood collection (its amount and frequency). It also contains information seeking consent to store the left-over specimen and publication of anonymized data.

### Interventions

#### Explanation for the choice of comparators {6b}

We will be using the generic sofosbuvir/velpatasvir combination, available in India, generic) for 12 weeks as a comparator because this is the undisputed standard of care and is recommended by all the international guidelines for HCV treatment. Sofosbuvir/velpatasvir was originally manufactured by Gilead Sciences (USA) and marketed under the brand name of ‘Epclusa’. Gilead Sciences have permitted several Indian companies to market its generic versions in the country and India has access to generics of sofosbuvir/velpatasvir manufactured by Indian companies such as Mylan Pharmaceutical Pvt Ltd (Anantpur, Hyderabad, India), Natco Pharma Ltd. (Kokjhar, Kamrup (R), Assam, India), Cadila Healthcare Limited (Kumrek, East-Sikkim, India) etc.

#### Intervention description {11a}

Participants in the intervention arm will be treated with sofosbuvir/velpatasvir combination (Indian generic) for 8 weeks. The drugs will be dispensed in plastic bottles containing 28 tablets each. On starting the treatment, only one bottle (28 tablets) will be dispensed and the participants will be followed at any time between 25–28 days of start of treatment. On 28 days follow-up, one bottle (for intervention arm) or two bottles (comparator arm) will be dispensed and the participants will be followed for SVR12.

#### Criteria for discontinuing or modifying allocated interventions {11b}

Patients can leave the study at any time for any reason if they wish to do so without any consequences. This study will be prematurely ended in case of any abundance in adverse events.

Criteria for study termination include any suspected unexpected serious adverse reaction or serious adverse event based on an allergic reaction and clear allergic or iatrogenic effects in two or more patients. Though we do not expect any serious adverse effect because we are neither using a new drug nor altered the dose or route of administration. We have only reduced the treatment duration.

#### Strategies to improve adherence to interventions {11c}

We have adopted the following mechanisms to improve the adherence and timeliness in follow-up (i) bottles of the drug will be labelled with their date for the next visit and contact number (ii) a trial coordinator will be in touch with all the participants till they have completed their follow-up (iii) empty bottles will be collected at the time of follow-up (iv) a call will be given, by the trial co-ordinator to the study participants, about a weeks before each of their planned visit.

#### Relevant concomitant care permitted or prohibited during the trial {11d}

All the drugs, which are safe to co-administer with sofosbuvir/velpatasvir and do not have significant drug to drug interaction, will be permitted to use during HCV treatment. If co-administration of acid suppressive medications is needed, we will prefer to use omeprazole at a dose of 20 mg once daily and sofosbuvir/velpatasvir would be administered with food 4 hours before omeprazole. For all the unavoidable concomitant medication, drug to drug interaction will be checked from the website maintained by University of Liverpool (https://www.hep-druginteractions.org/checker).

#### Provisions for post-trial care {30}

All those who will fail to achieve SVR12 in 8 weeks arm will be retreated with a repeat course of 12 weeks therapy. Retreatment will be provided free of cost.

### Outcomes {12}

**Primary outcome measure.** The proportion of participants who have undetectable HCV RNA after 12 weeks of stopping the antiviral drugs (SVR12). Quantitative assay of HCV RNA will be done using COBAS® AmpliPrep/COBAS® TaqMan® HCV quantitative Test, v2.0 (Roche, Branchburg, NJ, USA), with the lower limit of detection of 15 IU/mL. We have chosen SVR12 as a primary outcome measure because it has been widely accepted as a standard measure to define the success of HCV treatment. We will primarily do a per-protocol analysis.

**Secondary outcome measure.** The proportion of participants who complete planned 8 weeks or 12 weeks of treatment. We have selected this outcome to know whether the shorter course of treatment will improve adherence to anti-HCV treatment.

### Participant timeline {13}

**Fig 1:**
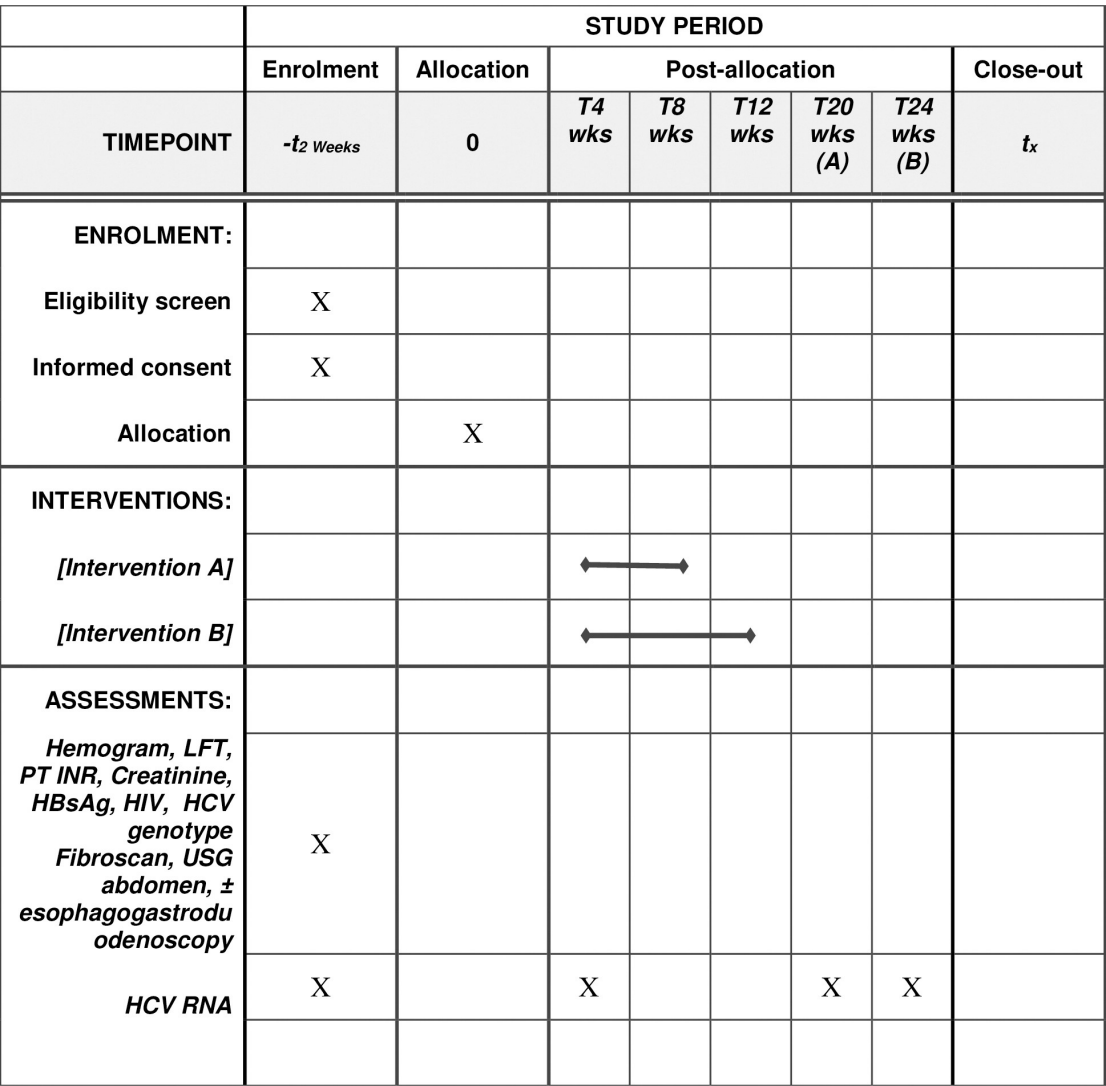
Proposed time line for the enrolment, interventions, and assessment of the participants in study.

The proposed timeline is summarized in Fig 1.

### Sample size {14}

We assumed the virological response in the reference group (12 weeks) is 95% and in the experiment group (8 weeks) as 90%, with a non-inferiority margin of 5%, power 90% and level of significance 2.5%, with a one-tailed alternative hypothesis. The required sample size is estimated as 400 participants in each arm. Assuming a 10% loss to follow-up, we raised the estimated sample size to 440 participants in each arm and a total study size of 880. We calculated sample size for per-protocol analysis.

### Recruitment {15}

We will be actively looking for HCV-positive patients visiting our OPD. All the participating centres are high-volume centres and likely enrol the required number of participants without much difficulty.

## Assignment of interventions: Allocation

### Sequence generation {16a}

Computer-generated sequence, block randomization, a block size of 4, 6, and 8, 1:1 ratio of allocation in two arms.

### Concealment mechanism {16b}

The sequence will be generated by the statistician (AA) and the generated sequence will be known to him only. Each of the generated sequence will be given a unique code. Participants in either of the five institute will have access to the serially listed unique codes but not the allocated treatment arm.

### Implementation {16c}

The allocation to a treatment arm in five canters will be coordinated between the trial coordinator, study centre investigator, and the statistician through WhatsApp or another similar medium. All the information about the patient’s enrolment will be kept updated on excel sheet shared via google drive. Once a patient is found eligible and consented to participation in any centre, the study site PI will ask for the treatment arm by sharing the next available unique code. The treatment arm will be disclosed by the statistician.

## Assignment of interventions: Blinding

### Who will be blinded {17a}

It is an open label study and no one will be blinded after the allocation of treatment arm.

### Procedure for unblinding if needed {17b}

Not applicable for our study.

## Data collection and management

### Plans for assessment and collection of outcomes {18a}

All the data will be captured in a predefined data collection form (DCF). The outcome will be assessed with serological testing of quantitative HCV RNA assay. Blood specimen collected in Co-PI’s centres will be stored, at -80° C and transported periodically to the PI’s institute (Sanjay Gandhi Postgraduate Institute of Medical Sciences). All the serological testing (HCV RNA and HCV genotyping) will be done in SGPGI only. The HCV RNA will be measured in serum with a standard commercially available quantitative assay with a lower limit of detection at <15 IU/mL.

HCV genotyping will be done with two-step, in -house method.

### Plans to promote participant retention and complete follow-up {18b}

We have adopted the following mechanisms to improve the adherence and timeliness in follow-up (i) bottles of the drug will be labelled with their date for next visit and contact number (ii) a trial coordinator will be in touch with all the participants till they have completed their follow-up (iii) a phone call will be given, by the trial coordinator to the study participants, about a weeks before each of their planned visit.

For those who were lost to follow-up, we will contact them on the phone on at least five different days over a period of 30 days.

### Data management {19}

All the data will be captured in a predefined data collection form (DCF). The data will be originally entered in a physical DCF. The research staff of each centre will be trained about screening, enrolment, consent, blood sample collection, and storage before the enrolment of the first participant from their centre. Scanned copy of the DCF will be shared with the trial coordinator. Each centre will enter the data from DCF to a common excel sheet which will be shared via google drive. The data, entered in the excel sheet, will be cross-checked by the trial coordinator. Trial coordinator will also monitor the follow-up of the study participants as well as the collection of blood specimen. The trial coordinator will also contact the participants from all the five participating centres to verify and cross-check the data.

### Confidentiality {27}

Research data will be stored using each participant’s identification details. The data will be started in form of physical copy of the data collection as well as in excel sheet. The data will be kept protected and assessable to the PI (Dr Amit Goel), four Co-PI, and the statistician only. Dall the relevant document will be stored for seven years according to the research guidelines after the study is completed. Details of the patient’s identity will not be reported in the publication.

### Plans for collection, laboratory evaluation and storage of biological specimens for genetic or molecular analysis in this trial/future use {33}

In addition to the PI’s institute (SGPGI), four other participating centres will also collect blood specimens from each participant. The blood specimens will be collected at baseline, at 4 weeks after start of treatment and for SVR12. Blood specimen collected in Co-PI’s centres will be stored, at -80° C, after proper labelling and serum separation. The stored specimen will be transported in dry ice, periodically to the PI’s institute (SGPGI). All the serological testing (HCV RNA and HCV genotyping) will be done in SGPGI only. The HCV RNA will be measured in serum with a standard commercially available quantitative assay which will have lower limit of detection at <15 IU/mL.

HCV genotyping will be done with two-step, in -house method. In brief, HCV RNA will be extracted using QIAamp Viral RNA Mini Kits (QIAGEN, Hilden, Germany) from serum and subjected to reverse-transcription using high-capacity cDNA RT Kit with RNAse inhibitor (Thermo Fisher Scientific, Massachusetts, United States) followed by amplification of DNA with PCR using primers corresponding to 5’UTR and NS5B regimens. The amplification products will be cleaned and subjected to Sanger sequencing using the BigDye Terminator version 3.1 dye chemistry on an ABI 3130 Genetic Analyzer (Applied Biosystems, Foster City, CA, USA) in both directions. The merged sequences will be aligned with reference sequences of various HCV genotypes retrieved from the GenBank database [http://www.ncbi.nlm.nih.gov/nucleotide], and a phylogenetic tree will be generated using MEGA 7 software and UPGMA method to identify the genotype.

The left-over sera will be stored at -80° C for future studies, if any.

## Statistical methods

### Statistical methods for primary and secondary outcomes {20a}

Categorical data will be expressed as proportion and ratio. Numerical data will be checked for nature of distribution and will be expressed as either mean±SD (if normally distributed) or median with interquartile range (if not normal distributed). The groups will be compared using non-parametric test of significance. The level of significance will be kept at p<0.05.

### Interim analyses {21b}

One interim analyses of the data will be done when the 12-week follow-up of the 50% of the required sample size is completed. If the proportion of participants who have achieved SVR12, between the two arms differ by more than 10%, then the study will be considered for pre-mature termination. The results will be communicated to the institute’s research committee. Their permission will be taken before the continuation of the study. We will use O’Brien and Fleming approach to conserve alpha in the interim analysis [18].

### Methods for additional analyses (e.g. subgroup analyses) {20b}

We will attempt subgroup analysis based on (i) HCV genotype 3 versus non-3 genotype and (ii) HCV viral load <6.0 million IU/mL versus ≥6 million IU/mL.

### Methods in analysis to handle protocol non-adherence and any statistical methods to handle missing data {20c}

The primary analysis will be per protocol analysis and will be aimed to include all participants in the arm they were originally allocated to. Missing value imputation will be used to handle missing data using all available baseline information. Sensitivity analyses will be performed to investigate the potential impact of missing data by using a pre-specified conservative multiple imputation strategy and a complete case analysis. We will do imputation only if more than 10% of values are missing [19].

### Plans to give access to the full protocol, participant level-data and statistical code {31c}

Full-protocol and patient level data can be accessed from the PI (AG). The access will be provided after obtaining necessary approval from the institute’s ethic committee.

## Oversight and monitoring

### Composition of the coordinating center and trial steering committee {5d}

The trial will be coordinated by the PI who is working in Sanjay Gandhi Postgraduate Institute of Medical Sciences, Lucknow, India. The entire trial will be supervised by the PI and coordinated by the trial coordinator, who will also be stationed in Sanjay Gandhi Postgraduate Institute of Medical Sciences.

### Composition of the data monitoring committee, its role and reporting structure {21a}

We do not have any data monitoring committee. The data will be supervised by the PI.

### Adverse event reporting and harms {22}

Data on all the self-reported adverse effects (AE) will be collected. The severity of AE will be graded from 1 to 5 according to the criteria laid by Common Terminology Criteria for Adverse Events (CTCAE) v5.0 [20]. Any AE of grade 3 or more will be reported as severe adverse effect (SAE) and will lead to discontinuation of drugs. Though we do not expect any adverse events because we are neither using a new drug nor altered the dose or route of administration. We have only reduced the treatment duration.

### Frequency and plans for auditing trial conduct {23}

We have no plan for auditing of trial conduct. Though, the trial will be constantly monitored by the PI.

### Plans for communicating important protocol amendments to relevant parties (e.g. trial participants, ethical committees) {25}

Any amendment in protocol will be communicate din writing to both, the institute etic committee and the funding agency.

## Dissemination plans {31a}

The results of the study will be communicated in form of report to the institute ethic committee of all the five participating institutes, and the funding agency. A detailed manuscript will be prepared and will be submitted for publication. The results of the study will not be informed to the study participants. There is no restriction by the funding agency on publication. Rather any publication will be appreciated by the funder.

## Discussion

This study will compare the effectiveness of 8 weeks of anti-HCV treatment with that of standard of care, i.e, 12 weeks of treatment. Being the first RCT to compare 8 weeks versus 12 weeks off treatment, we restrict to include non-cirrhotic patients only. If the outcome of the two arms is found to be comparable, it may prove a milestone in reducing the treatment duration as well as the cost of the treatment. Our results may have global implications.

## Supporting information

S1 Checklist SPIRIT checklist. (DOC)

S1 File Study protocol submitted for ethic clearance. (DOC)

## Abbreviations

APRI: AST platelet ratio
DAA: Direct acting antivirals
FIB-4: FIB-4
HCV: Hepatitis C virus
PID: Patient information document
RNA: Ribonucleic acid
RVR4: Rapid virological response at 4 weeks
SVR12: Sustained virological response at 12 weeks after treatment
