# Supplementary material for: Eight versus twelve weeks of sofosbuvir-velpatasvir in treatment-naïve non-cirrhotic patients with chronic hepatitis C virus infection: Study protocol for a multicentric, open labelled, randomized, non-inferiority trial (RESOLVE trial)
Source: PLoS One. 2023 May 18;18(5):e0285725. doi: 10.1371/journal.pone.0285725 (PMC10194938; doi:10.1371/journal.pone.0285725)
Supplement: S1 File — (DOC) [file pone.0285725.s002.doc]

**RE**duced duration of **E**ight versus twel**V**e weeks of **SO**fosbuvir-Ve**L**patasvir in treatment naive non-cirrhotic patients with chronic hepatitis C virus infection: A Multicentric, Open labeled, Randomized, Non-inferiority trial **(RESOLVE)**

**Review of existing knowledge related to proposed research proposal**

*Hepatitis C virus (HCV)*

Hepatitis C (HCV) virus is a hepatotropic virus wwhich infects and multiply in hepatocytes. A large proportion of those who acquires HCV infection, fails to clear the virus naturally and progress to develop chronic hepatitis C (CHC) (3). Unchecked CHC, in a sizeable proportion, may progress to liver cirrhosis and hepatocellular carcinoma (4, 5), both of which are potentially-fatal conditions. All those with CHC shall be treated to clear the virus.

*Burden of HCV infections – worldwide*

World Health Organization’s (WHO) global estimates, viral hepatitis caused 1.34 million deaths in 2015. This number is comparable to deaths caused by tuberculosis and higher than those caused by HIV. Most of the viral hepatitis related deaths in 2015 were due to chronic liver disease (720,000 deaths due to cirrhosis) and primary liver cancer (470,000 deaths due to hepatocellular carcinoma). Globally, in 2015, an estimated 71 million people were living with chronic HCV infection (6) and it accounted for approximately 399,000 annual deaths.

*Global target of HCV elimination*

Considering the huge burden of HCV and its associated morbidity/mortality, the World Health Assembly in May 2016 endorsed the Global Health Sector Strategy (GHSS) for 2016–2021 on viral hepatitis (HBV and HCV infection), which proposes to eliminate viral hepatitis as a public health threat by 2030. The elimination is defined as reduction, as compared to baseline in 2015, in new infections (incidence) by 90% and viral hepatitis related deaths (mortality) by 65% by the year 2030.

To achieve the WHO target, the government of India has recently launched public funded “National Viral Hepatitis Control Program (NVHCP)”.

*HCV treatment*

HCV infection is treated with a group of drugs called as Direct Acting Antiviral agents (DAAs). These DAAs are orally administered and are highly safe and effective. The effectiveness of these drugs is slightly low in patients with cirrhosis. In absence of cirrhosis, these DAAs successfully clears the virus in ~95% of those who have taken the complete course of treatment (7).

DAAs based regimen are effective against either certain genotypes (genotype specific regimen) or all genotypes (pangenotypic regimen). Use of genotype specific regimen need genotyping of the virus circulating in a given patient before start of DAA. HCV genotyping is a costly investigation which need time, trained manpower, and specific instruments. Hence, across the globe, pangenotypic treatment are preferred over genotypic specific regimens. At least six genotypes are known for HCV, namely HCV genotypes 1-6. In India, the HCV genotype 3 and 1 are the most common and account for more than 95% of HCV infection (8).

Several genotypic specific and pangenotypic DAAs based treatment regimens are approved for the treatment of non-cirrhotic patients with genotype 3 or genotype 1 HCV infection. Among them, only few are available in India (Table 1) (9).

**Table 1: Treatment regimens available in India**

| **No** | **Type** | **Genotypes against which it is effective** | **Drugs** | **Duration** |
| --- | --- | --- | --- | --- |
| 1 | Genotype specific | Genotype 1, 4, 5, 6 | Sofosbuvir plus Ledipasvir | 12 weeks |
| 2 | Genotype specific | Genotype 3 | Sofosbuvir plus Daclatasvir | 12 weeks |
| 3 | Pangenotypic | All genotypes | Sofosbuvir plus Velpatasvir | 12 weeks |

*Comparison of 8 weeks versus 12 weeks of HCV treatment*

One of the major bottle-neck in successful HCV treatment is “12 weeks duration” of treatment. Long-duration of treatment poses logistic issues of maintaining the drug supply, poor drug compliance, and increased cost of therapy. Hence, efforts are done to reduce the treatment duration without compromising its effectiveness. Several studies, for the regimens which are available in India, have shown that the 8 weeks of treatment duration are comparable to usual 12 weeks of therapy (Table 2).

**Table 2: Data on comparison of 8 weeks versus 12 weeks of treatment regimens (available in India) in non-cirrhotic patients with HCV infection**

| **No** | **Author, year** | **Patient characteristics** | **Study design** | **DAA Combination used** | **Virological response as SVR12 (%)** | |
| --- | --- | --- | --- | --- | --- | --- |
|  |  |  |  |  | **8 weeks** | **12 weeks** |
| 1 | Kowdley et al, 2014 (ION-3) (10) | Non-cirrhotic, Genotype 1, treatment naive | Open-label, RCT | Sofosbuvir plus Ledipasvir | 94 | 95 |
| 2 | Marcus et al, 2018 (11) | Non-cirrhotic, Genotype 1, treatment naive | Open-label, RCT | Sofosbuvir plus Ledipasvir | 96.3 | 96.3 |
| 3 | Shiha et al, 2019 (12) | Non-cirrhotic, Genotype 4, treatment naive | Open-label, RCT | Sofosbuvir plus Ledipasvir | 95 | 98 |
| 4 | Yanny et al, 2018 (13) | Geriatric population, non-cirrhotic, genotype 1 | Retrospective study | Sofosbuvir plus Ledipasvir | 93 | 95 |
| 5 | Boyle et al, 2020 (14) | F2 or F3 fibrosis, Genotype 3, Treatment naive | Single arm (8 weeks duration), Observational study | Sofosbuvir plus Velpatasvir | 100  (per protocol analysis) | |
| 5 | Fu et al, 2021 (15) | Adolescent and children with HCV infection | Systematic review of 36 studies | Various combination | 100 | 98.8 |

*HCV burden in India*

Recently, our group conducted a systematic review of all the published literature on prevalence of HCV infection in India (16). This document from the basis of disease burden estimation for the “National Viral Hepatitis Control Program (NVHCP)” of India. In this review, we extracted data from 422 data points included in 355 studies. Among low-risk group population, we found that the anti-HCV seroprevalence in the country was anti-HCV seroprevalence rates was 0.85% (95% CI: 0.00%-3.98%) in community-based studies and 0.44% (0.40-0.49) in blood donors.

These data suggest that anti-HCV antibody prevalence in the Indian population is likely to be between 0.4% and 1.0%. Applying this to current population of India of around 1.30 billion, the number of anti-HCV positive persons in the country is thus likely to be between 5.2 and 13 million. With the prevalence of viremia among anti-HCV seropositive persons of 60%-70%, the number of persons with active HCV infection in the country should be in the range of ~3.0 to 9.0 million (16).

This systematic review is recently updated by our group to extract more accurate and updated information on HCV seroprevalence in the country (Unpublished report).

*Cost-effectiveness of HCV treatment in India*

Our previous work on mathematical modelling has shown that the treatment of HCV at a wider scale will be cost-effective as well as cost saving (17). This work supported the Government of India in planning the NVHCP. Our previous work had shown that the use of sofosbuvir/velpatasvir available at the current cost in the country is equal to sofosbuvir/daclatasvir (18). The cost-effectiveness will further be increased if the treatment duration could be reduced from 12 weeks to 8 weeks.

*National Viral Hepatitis Control Program (NVHCP) of India*

Government of India has launched a public funded, nationwide NVHCP. Though the program aims at prevention and control of all the viral hepatitis (Hepatitis A, Hepatitis B, Hepatitis C, and Hepatitis E) but as of now it is focussing on control and treatment of HCV. The national guidelines for the laboratory testing, diagnosis, and treatment are already in place and the treatment has been started in a few centres.

**Research gap:**

- Whether the duration of HCV treatment can be reduced without compromising the efficacy?

**Hypothesis**

Treatment with eight weeks of sofosbuvir-velpatasvir combination will not be inferior to that with twelve weeks in non-cirrhotic patients with chronic HCV infection.

**Objective of the study**

*Primary objective*

To compare the sustained virological response at week 12 (SVR12) after stopping 8 weeks or 12 weeks of sofosbuvir/velpatasvir in non-cirrhotic patients with chronic hepatitis C virus infection

*Secondary objective*

To compare the proportion of participants completing their planned 8 weeks or 12 weeks of treatment

*Relevance of proposed research project to public health*

If the 8 weeks treatment is proven to be non-inferior to usual 12 weeks treatment, then a shorter treatment duration can be widely implemented in national viral hepatitis control program. A shorter course of treatment is likely to have the following benefits

- Use of a single drug combination, instead of two different drugs, will ease the implementation of NVHCP in the most remote areas of the country without increasing the cost of treatment.
- Reduced treatment duration is likely to enhance the patients’ compliance to the drug treatment
- Enhanced compliance will culminate in reduced risk for drug default and drug resistance
- The results of this study could also be applied to several other Asian countries where the disease burden is high and the drug availability are similar to that in India.

*Relative importance of proposed research project in a given area (100 words)*

World Health Organization (WHO) has set the global target to eliminate the HCV by the year 2030 (1). The HCV elimination can be achieved through focus on prevention of new infection and treatment of infected persons. To achieve this target, India has recently launched a nationwide, public funded “National Viral Hepatitis Control Program (NVHCP)”.

In present era, HCV is treated with oral drugs which are safe and highly effective. Several treatment regimens are approved for HCV treatment. All the DAA regimes, which are available in India, has to be given for 12 weeks.

One of the major bottle-neck in successful HCV treatment is “12 weeks duration” of treatment. Long-duration of treatment poses logistic issues of maintaining the uninterrupted drug supply by the treatment provider and poor drug compliance by the patient. Hence, efforts are done to reduce the treatment duration without compromising its effectiveness.

*Applicability of your research project*

*(a) Short term*

HCV infected patients could effectively be treated with a shorter course of drugs.

*(b) Long term*

The results of this study and cost-effectiveness analysis are likely to lay down better policies as a part of the National Viral Hepatitis Control Program (NVHCP) of India, and thus should have a long-term, country-wide application. If the treatment duration could be reduced it will save money, improve the drug compliance, and increase the chance for the success of HCV elimination through NVHCP in the country.

**Preliminary work already done by the Investigator on this problem, e.g. selection of subjects, standardization of methods, with results, if any.**

We have treated a group of 27 dialysis dependent patients with acute hepatitis C virus infection. They were treated, regardless of their HCV genotype, with half daily dose of sofosbuvir plus daclatasvir for 8 weeks. The sustained virological response at 12 weeks (SVR12) on per protocol analysis was 96%. The manuscript has been published in a journal of Impact factor 6.0. The results of this study indicate that a shorter 8-week course of anti-HCV drugs may be as effective as the usual 12 weeks course.

***Goel A****, Bhadauria DS, Kaul A, Verma A, Tiwari P, Rungta S, Rai P, Gupta A, Aggarwal R. Acute hepatitis C treatment in advanced renal failure using 8 weeks of pan-genotypic daclatasvir and reduced-dose sofosbuvir. Nephrol Dial Transplant. 2020 Oct 23*

**Detailed research plan:**

*Methodology*

*Study design:* Multicentric, Open label, Randomized, Non-inferiority trial

*Study centres:*

1. Department of Gastroenterology, Sanjay Gandhi Postgraduate Institute of Medical Sciences, Lucknow (PI)
2. Department of Gastroenterology, King George’s Medical University, Lucknow
3. Department of Gastroenterology, Institute of Medical Sciences, Banaras Hindu University, Varanasi
4. Department of Gastroenterology, All Indian Institute of Medical Sciences, New Delhi
5. Department of Gastroenterology, GB Pant Hospital, New Delhi

*Study duration:* Three years: July 2022-June 2025

*Participant selection:* Participants who are attending the outpatient services in either of the participating institutes will be prospectively enrolled.

*Sequence generation:* central sequence generation will be done at PI’s institute (SGPGI) and the participants in either of the five institute will be sequentially allocated to the study arms. All the five centres will be coordinated by trial coordinate through WhatsApp or other similar medium.

*Randomization:* Computer generated sequence, Block randomization, block size of 4, 6 or 8, 1:1 ratio of allocation in two arms.

*Screening criteria*

All adults with active HCV infection will be screened for selection criteria

*Inclusion criteria*

1. Age >18 years
2. HCV mono-infection
3. Detectable HCV RNA (>10,000 IU/mL) in serum
4. Chronic HCV infection
5. No evidence of cirrhosis based upon a combination of history, examination, ultrasound, Transient elastography, APRI, FIB-4, Upper GI endoscopy

Exclusion criteria

1. Presence of cirrhosis
2. HBsAg or HIV coinfection
3. Estimated GFR <30 ml/ minute
4. Prior exposure to oral anti-HCV drugs, i.e, DAAs
5. Hepatocellular carcinoma or any other malignancy
6. Portal vein thrombosis
7. High-risk population such as people living with HIV, people on maintenance hemodialysis, thalassaemic or haemophiliacs, people who inject drugs, men have sex with men, high risk sexual behaviour

*Cirrhosis*

Cirrhosis will be defined as summarized in Figure 1 (given below)


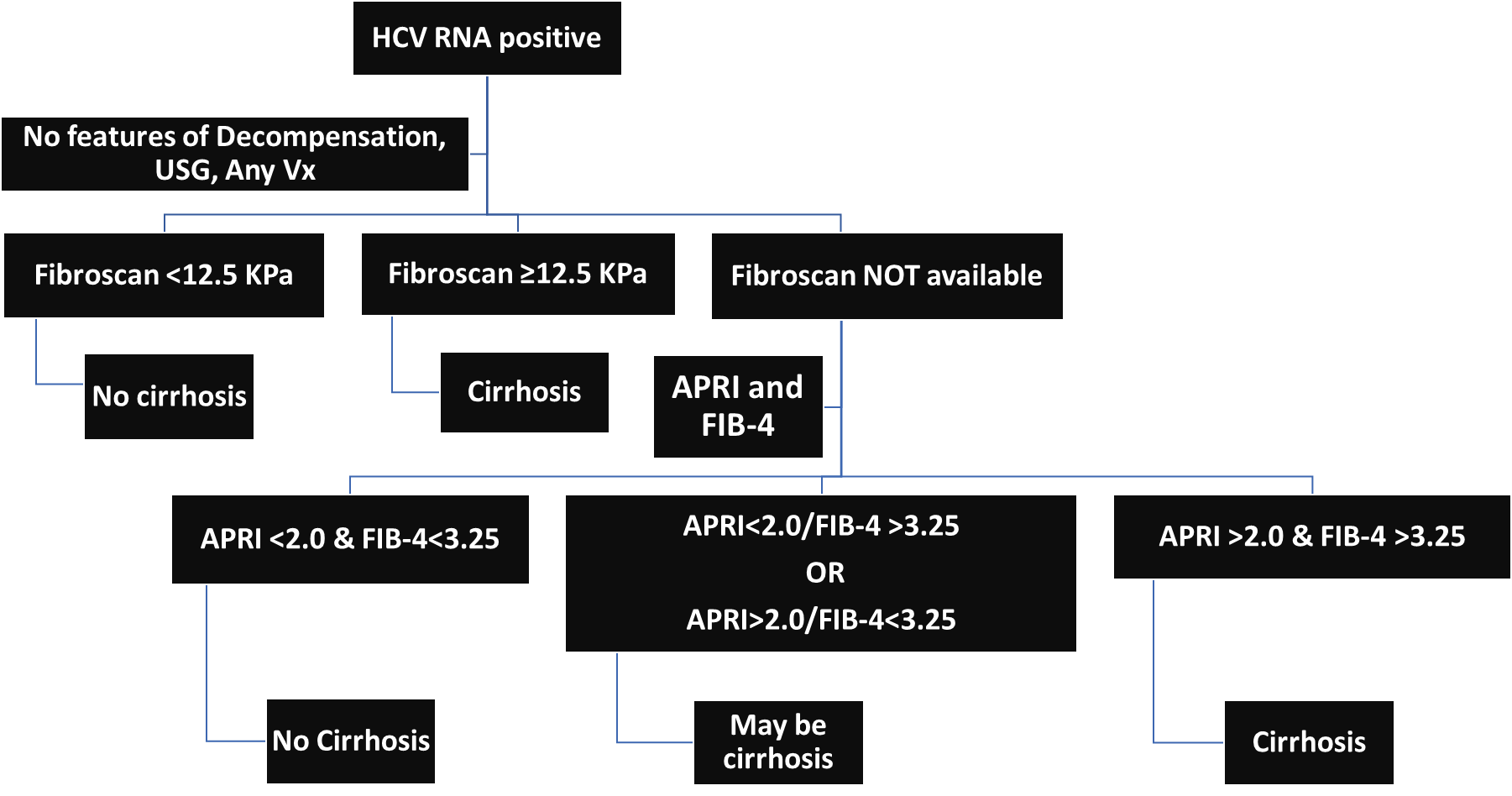


**Flow of the participants**


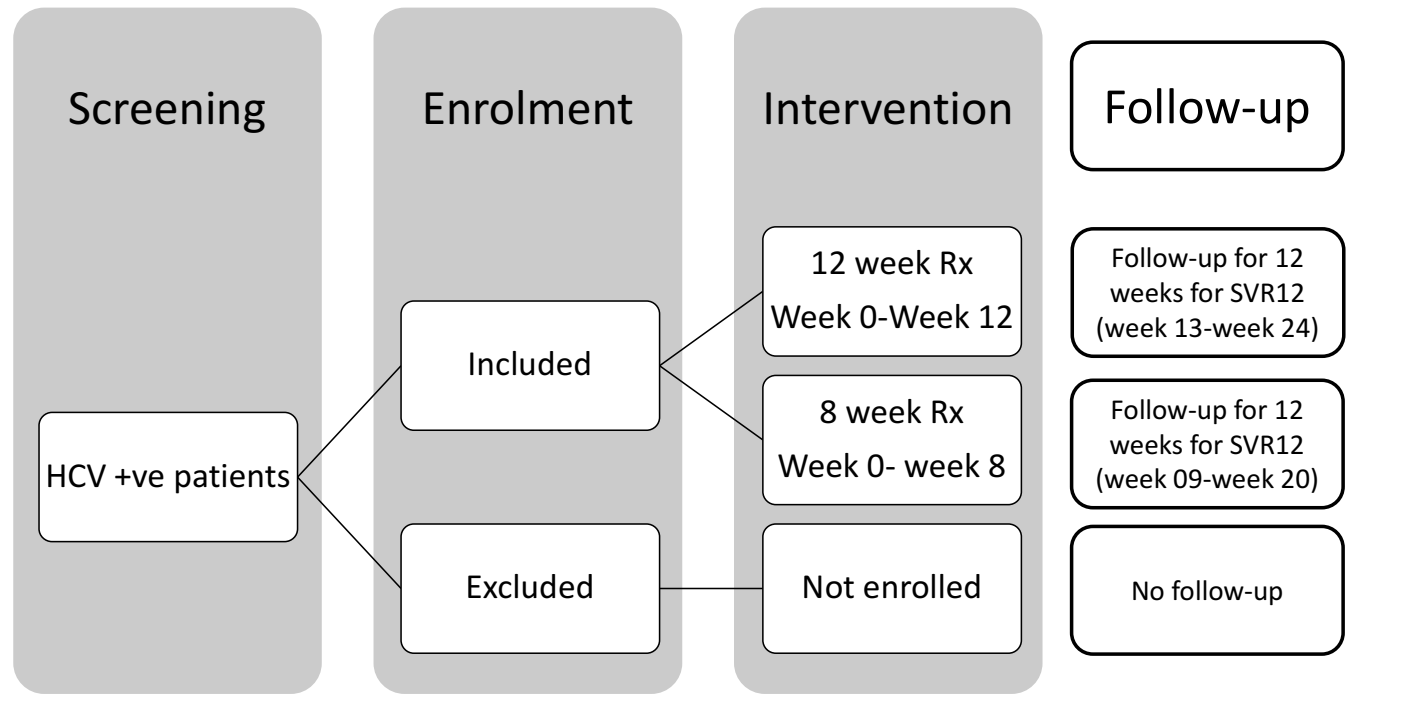


*Sample size:*

We assumed the virological response in the reference group (12 weeks) is 95% and in the experiment group (8 weeks) as 90%, with a non-inferiority margin  of 5%, power 90% and level of significance 2.5%, with a one tailed alternative hypothesis. The required sample size is estimated as 400 participants in each arm. Assuming a 10% loss  to follow up, we raised the estimated sample size to 440 participants in each arm and total study size of 880.

**Study arms**

Two arms

**(i) Standard of care arm**: participants will receive 12 weeks of sofosbuvir/velpatasvir combination

**(ii) Intervention arm**: participants will receive 8 weeks of sofosbuvir/velpatasvir combination

**Study end points**

The study will end if any of the following end point have been achieved by the study participant

1. Twelve weeks of follow-up completed after treatment completion
2. Death/lost to follow-up at any time from enrolment in study to completion of 12 weeks of follow-up
3. Detectable HCV RNA at any time point after completion of 8 or 12 weeks of DAA treatment
4. Drug discontinuation for more than two weeks at any time during treatment administration

*Follow-up of the participants*

All the participants will be followed at 4 weeks, end of treatment (8 weeks or 12 weeks of treatment) and at 12 weeks after stopping the treatment.

*Specimen collection*

A blood specimens will be collected before starting anti-HCV treatment. The blood specimens, collected before starting DAA, will be tested for HCV genotype. Genotyping will be done after the planned treatment is completed. All genotyping will be done in SGPGI only and blood specimens will be anonymised at the time of genotyping. The results of genotyping will not be revealed to the investigators till the end of the study and data are analyzed.

We will also attempt our best to collect another blood specimen after 4 weeks of anti-HCV treatment. The collected blood specimen will be transported to SGPGI for quantitative HCV RNA assay to study the rapid virological response at 4 weeks (RVR4).

*Methods for HCV genotyping*

RNA will be extracted from HCV RNA containing serum specimens using a QIAamp viral RNA Mini Kit (QIAGEN GmBH, Germany). Extracted RNA will be reverse transcribed using High-Capacity cDNA Reverse Transcription Kit (Thermo Fischer Scientific, United States) which uses the random primer scheme for initiating cDNA synthesis. For amplification and genotyping, primers specific for HCV 5’-UTR (forward primer 5’ATGGATCACTCCCCTGTGAGGAACT3’, reverse primer 5’GTCTACGAGACCTCCCGGGGCACT3’) and NS5B regions (forward primer 5’ACYACCATYATGGCNAARARYGAGGT3’, reverse primer 5’TAYCTRGTCATRGCCTCCGTGAAGRC3’) will be used. Amplification will be confirmed using 2% gel electrophoresis. The amplified product will be cleaned up to remove excess primers and unincorporated nucleotides, and will then be subjected to Sanger sequencing in both directions using the BigDye Terminator version 3.1 dye chemistry and on an ABI 3130 Genetic Analyzer (Applied Biosystems, Foster City, CA, USA). The sequences obtained in the two directions will be merged. The merged sequences for individual specimens will be aligned with each other and with those for various HCV genotypes retrieved from GenBank database [ http://www.ncbi.nlm.nih.gov/nucleotide ]). A phylogenetic tree will be constructed using MEGA 7 software and UPGMA method for the sequences obtained.

*Interim analysis*

One interim analyses of the data will be done when the 12-week follow-up of the 50% of the required sample size is completed. The results will be communicated to the institutes research committee.

*Significance/outcome of the proposed work.*

If the 8 weeks treatment is proven to be non-inferior to usual 12 weeks treatment, then a shorter treatment duration can be widely implemented in national viral hepatitis control program. A shorter course of treatment is likely to have the following benefits

- It will ease the implementation of NVHCP in the country.
- Reduced treatment duration is likely to enhance the patients’ compliance and the rate of treatment completion
- Reduced treatment duration is likely to reduce the cost of HCV treatment

**Proposed time schedule for stud**y

| **Activity** | **Time duration** | **Total time duration** |
| --- | --- | --- |
| Procurement and recruitment | 3 months | 3 months |
| Recruitment of the participants | 22 months | 25 months |
| Last follow-up of the participants | 6 months | 31 months |
| Data analysis, writing the final reports | 5 months | 36 months |

**Retreatment of those who relapse after 8 weeks of sofosbuvir/velpatasvir combination**

All such participants will be retreated, free of cost, with 12 weeks of sofosbuvir/velpatasvir combination. Though their retreatment results will not be considered in our analysis.

**References**

1. World Health Organization (WHO). Global health sector strategy on viral hepatitis 2016-21; 2016.
2. National Viral Hepatitis Control Program, Ministry of Health, Government of India. National guidelines for diagnosis and management of viral hepatitis 2018
3. Hajarizadeh B, Grebely J, Dore GJ. Epidemiology and natural history of HCV infection. Nature reviews Gastroenterology & hepatology. 2013;10(9):553-62.
4. Perz JF, Armstrong GL, Farrington LA, Hutin YJ, Bell BP. The contributions of hepatitis B virus and hepatitis C virus infections to cirrhosis and primary liver cancer worldwide. Journal of hepatology. 2006;45(4):529-38.
5. Seeff LB. Natural history of chronic hepatitis C. Hepatology (Baltimore, Md). 2002;36(5 Suppl 1):S35-46.
6. World Health Organization (WHO). Global Hepatitis Report. 2017
7. Kohli A, Shaffer A, Sherman A, Kottilil S. Treatment of hepatitis C: a systematic review. Jama. 2014;312(6):631-40.
8. Narahari S, Juwle A, Basak S, Saranath D. Prevalence and geographic distribution of Hepatitis C Virus genotypes in Indian patient cohort. Infection, genetics and evolution : journal of molecular epidemiology and evolutionary genetics in infectious diseases. 2009;9(4):643-5.
9. HCV Guidance: Recommendations for Testing, Managing, and Treating Hepatitis C (www.hcvguidance.org) as accessed on 21^st^ November 2020
10. Kowdley KV, Gordon SC, Reddy KR, Rossaro L, Bernstein DE, Lawitz E, et al. Ledipasvir and sofosbuvir for 8 or 12 weeks for chronic HCV without cirrhosis. The New England journal of medicine. 2014;370(20):1879-88.
11. Marcus JL, Hurley LB, Chamberland S, Champsi JH, Gittleman LC, Korn DG, et al. No Difference in Effectiveness of 8 vs 12 Weeks of Ledipasvir and Sofosbuvir for Treatment of Hepatitis C in Black Patients. Clinical gastroenterology and hepatology : the official clinical practice journal of the American Gastroenterological Association. 2018;16(6):927-35.
12. Shiha G, Esmat G, Hassany M, Soliman R, Elbasiony M, Fouad R, et al. Ledipasvir/sofosbuvir with or without ribavirin for 8 or 12 weeks for the treatment of HCV genotype 4 infection: results from a randomised phase III study in Egypt. Gut. 2019;68(4):721-8.
13. Yanny, B., Saab, S., Durazo, F. et al. Eight-Week Hepatitis C Treatment with New Direct Acting Antivirals Has a Better Safety Profile While Being Effective in the Treatment-Naïve Geriatric Population Without Liver Cirrhosis and Hepatitis C Virus-RNA < 6 Million IU/mL. Dig Dis Sci 2018; 63: 3480–3486
14. Boyle A, Marra F, Peters E, Datta S, Ritchie T, Priest M, et al. Eight weeks of sofosbuvir/velpatasvir for genotype 3 hepatitis C in previously untreated patients with significant (F2/3) fibrosis. Journal of viral hepatitis. 2020;27(4):371-5.
15. Fu Z, Dong C, Ge Z, Wang C, Zhang Y, Shen C, Li J, Zhu C, Wang Y, Huang P, Yue M. High SVR12 With 8-Week Course of Direct-Acting Antivirals in Adolescents and Children With Chronic Hepatitis C: A Comprehensive Analysis. Front Med (Lausanne). 2021 Jun 8;8:608760.
16. **Goel A**, Seguy N, Aggarwal R. Burden of hepatitis C virus infection in India: A systematic review and meta-analysis. J Gastroenterol Hepatol. 2019 Feb;34(2):321-329
17. Aggarwal R, Chen Q, **Goel A**, Seguy N, Pendse R, Ayer T, Chhatwal J. Cost-effectiveness of hepatitis C treatment using generic direct-acting antivirals available in India. PLoS One. 2017 May 17;12(5): e0176503
18. **Goel A**, Chen Q, Chhatwal J, Aggarwal R. Cost-effectiveness of generic pan-genotypic sofosbuvir/velpatasvir versus genotype-dependent direct-acting antivirals for hepatitis C treatment. J Gastroenterol Hepatol. 2018 Dec;33(12):2029-2036

**Collaborators**

| No | Study centre | PI from the study centre | Roles | Contribution |
| --- | --- | --- | --- | --- |
| 1 | Department of Gastroenterology,  Sanjay Gandhi Postgraduate Institute of Medical Sciences  Lucknow, India | Dr Amit Goel  Additional Professor  agoel.ag@gmail.com  Phone: 8765974037 | PI | Overall supervision and coordination of the trial  Recruitment/follow-up of the participants  HCV genotyping |
| 2 | Department of Gastroenterology,  King George’s Medical University  Lucknow, India | Dr Sumit Rungta  Associate Professor  drsumitrungta79@gmail.com  Phone: 9935537944 | Co-PI | Recruitment/follow-up of the participants |
| 3 | Department of Gastroenterology,  Institute of Medical Sciences  Banaras Hindu University  Varanasi, India | Dr Vinod Kumar  Assistant Professor  drv_inod@yahoo.co.in  Phone: 9984719346 | Co-PI | Recruitment/follow-up of the participants |
| 4 | Department of Gastroenterology,  All India Institute of Medical Sciences,  New Delhi, India | Dr Shalimar  Additional Professor  drshalimar@gmail.com  Phone: 9968405815 | Co-PI | Recruitment/follow-up of the participants |
| 5 | Department of Gastroenterology  Govind Ballabh Path Hospital  New Delhi | Dr Ajay Kumar  Additional Professor  ajaykumar.aiims@gmail.com  Phone:9968147901 | Co-PI | Recruitment/follow-up of the participants |
| 6 | Centre for Chronic Conditions and Injuries  Public Health Foundation of India, Gurgaon, India | Dr Ashish Awasthi  INSPIRE Faculty  ashishbhuims@gmail.com  9208604604 | Collaborator | Data analysis |
